# The effect of the Ras homolog gene family (Rho), member A/Rho associated coiled-coil forming protein kinase pathway in atrial fibrosis of type 2 diabetes in rats

**DOI:** 10.3892/etm.2014.1843

**Published:** 2014-07-14

**Authors:** JINLING CHEN, QINGQING LI, RUIQING DONG, HUIKUAN GAO, HUI PENG, YONGQUAN WU

**Affiliations:** Department of Cardiology, Beijing Friendship Hospital, Capital Medical University, Beijing 100050, P.R. China

**Keywords:** atrial fibrosis, atrial arrhythmogenicity, fasudil, Ras homolog gene family, member A (RhoA)/Rho associated coiled-coil forming protein kinase pathway

## Abstract

Diabetes mellitus promotes atrial structural remodeling, thereby producing atrial arrhythmogenicity. Atrial arrhythmia can substantially increase the risk of premature death. The aim of this study was to investigate the role of Ras homolog gene family, member A (RhoA)/Rho associated coiled-coil forming protein kinase (ROCK) in atrial fibrosis in diabetic hearts, and the effects of fasudil hydrochloride hydrate on atrial fibrosis. An eight-week-old male Sprague-Dawley rat model of type 2 diabetes was established using a high-fat diet combined with streptozotocin [30 mg/kg, once, intraperitoneal (i.p.)]. Animals were randomly divided into three groups: Control rats, untreated diabetic rats that received vehicle, and treated diabetic rats that received Rho kinase inhibitor fasudil hydrochloride hydrate (10 mg/kg/day, i.p., for 14 weeks). The morphological features of atrial fibrosis were observed using Masson staining. The mRNA expression levels of RhoA, ROCK1, ROCK2, type-I and type-III procollagen were assessed with quantitative polymerase chain reaction. The protein levels of RhoA, ROCK1 and ROCK2 were evaluated using western blot analysis. The atria of untreated diabetic rats showed evident atrial fibrosis as compared to the control rats; the mRNA expression levels of RhoA, ROCK1, ROCK2, type-I and type-III procollagen were upregulated; and the protein levels of RhoA, ROCK1 and ROCK2 were increased. The treatment with fasudil hydrochloride hydrate significantly reduced atrial fibrosis, mRNA levels of RhoA, ROCK1, ROCK2, type-I and type-III procollagen, and the protein levels of RhoA, ROCK1 and ROCK2. The results suggested that RhoA/ROCK was involved in atrial fibrosis, and that fasudil hydrochloride hydrate ameliorates atrial fibrosis through the RhoA/ROCK pathway in rats with type 2 diabetes.

## Introduction

Diabetes mellitus (DM) could lead to fibrosis of multiple organs, including atrial structural remodeling (1). Atrial fibrosis, as a hallmark of atrial structural remodeling, plays a critical role in the occurrence and maintenance of atrial arrhythmogenicity. Fibrosis isolates groups of atrial and individual myocytes and thus impairs cell-to-cell coupling, leading to inhomogeneities in intra- and interatrial conduction and retarded conduction velocity. The mechanisms responsible for atrial fibrosis remain to be clarified. Currently, there are no specific drugs available for the prevention or treatment of atrial fibrosis in DM.

The Ras homolog gene family, member A (RhoA)/Rho associated coiled-coil forming protein kinase (ROCK) is a member of the serine/threonine kinase family. ROCK is the most studied Rho downstream effector. It has two known isoforms (ROCK1 and ROCK2) and regulates cytoskeletal reorganization by phosphorylating myosin phosphatase, which results in an increase in myosin light chain (MLC) phosphorylation (2). Through this, the RhoA/ROCK pathway is involved in regulating endothelial migration, platelet activation, thrombosis, and oxidative stress as well as smooth muscle contraction (3,4). A number of abnormal activations of the RhoA/ROCK pathway are present in diseases (5). It is activated by multiple cytokines and inflammatory mediators, including platelet-derived growth factor, transforming growth factor-β, endothelin-1 and angiotensin II (6). Previous studies have suggested that the RhoA/Rock pathway also plays a significant role in bowel (7), liver (8), renal (9–10), pulmonary (11), and myocardial fibrosis (12–13). Thus inhibition of this signaling pathway is effective for treating a wide range of cardiovascular and non-cardiovascular disease. Fasudil is a Rho-kinase inhibitor, and has previously been shown to attenuate fibrosis in multiple organs (9,11–12).

We hypothesized that the RhoA/ROCK pathway is involved in atrial fibrosis, and that fasudil, which is a highly selective inhibitor of the two ROCK isoforms, may inhibit the development of atrial fibrosis. Therefore, the present study focused on the expression and function of the RhoA/ROCK pathway in a rat model of type 2 diabetes.

## Materials and methods

The experimental protocol was approved by the Ethics Review Committee for Animal Experimentation of the Beijing Friendship Hospital (Beijing, China).

### Animal model

Eight-week-old male Sprague-Dawley rats, weighing 240–260 g, were purchased from Vital River Laboratories (Beijing, China). After two weeks of acclimatization, the rats were randomly assigned to receive a standard diet as the normal control group or a high-fat diet (HFD; 73% standard diet, 25% lard, and 2% yolk powder) for four weeks. At the end of the sixth week, control rats were injected intraperitoneally (i.p.) with vehicle (0.1 mol/l citric acid buffer) once and given a standard diet sequentially. HFD rats were injected i.p. with a low-dose (30 mg/kg) of streptozotocin (STZ; Sigma-Aldrich, St. Louis, MO, USA) and given an HFD sequentially. Blood glucose (BG) was measured in whole blood collected from the tail vein by a portable glucometer and BG levels were recorded every week. Rats with BG levels of >16.7 mmol/l and that were stable for four weeks were considered to be diabetic. From the 10th week, control rats remained on a standard diet and were treated with i.p. injection of sterile vehicle every day. Diabetic rats were randomly divided into two groups: i) Treated diabetic rats were maintained on a HFD and received fasudil hydrochloride hydrate (10 mg/kg/day; i.p.) (Tianjin Hongri Company, Tianjin, China) every day for 14 weeks; and ii) untreated diabetic rats were treated with i.p. injection of sterile vehicle every day for 14 weeks. At week 24, fasting plasma was collected for further measurement of fasting insulin (FINS) and fasting BG (FBG). Homeostasis model assessment for insulin resistance (HOMA-IR) was calculated as FBG x FINS/22.5 and was used in assessing insulin resistance. The rats were sacrificed and the hearts were harvested. A section of tissue from the left atrium was fixed in 4% paraformaldehyde and embedded in paraffin for histological examination. The remainder of the left and right atria were snap-frozen and stored at −80°C until processing for the extraction of mRNA and protein.

### Masson staining

The tissue samples were washed with water and then stained using the following steps: i) Fixated in Bouin’s solution, microwaved for 1 min, left to stand for 15 min; ii) washed under a running tap water to remove the picric acid for 5 min; iii) stained in Weigert’s hematoxylin working solution for 10 min; iv) waited for it to turn blue under running tap water for 5 min and then rinsed with distilled water; v) added Biebrich scarlet solution for 5 min; vi) rinsed with distilled water; vii) differentiated in phosphotungstic/phosphomolybdic acid solution for 10 min; viii) transferred directly into aniline blue solution for 5 min; ix) rinsed with distilled water; x) differentiated in 1% acetic acid for 1 min, rinsed with distilled water and xi) dehydrated, cleared and mounted on a coverslip.

### Isolation of RNA and quantitative polymerase chain reaction (qPCR)

Small sections of the atria were sampled. Total RNA was prepared from the right and left atrial free walls using TRIzol^®^ reagent according to the manufacturer’s instructions. The purity of isolated RNA was identified by ultraviolet spectrometry. cDNA was synthesized by reverse transcription using oligo dT (deoxythymine) primer and M-MLV (moloney murine leukemia virus) reverse transcriptase (Promega Corp., Fitchburg, WI, USA), which was used as a template in the subsequent PCR analysis. The mRNA levels of RhoA, ROCK1, ROCK2, collagen type-I and collagen type-III were evaluated by qPCR. The level of GAPDH was also evaluated as the internal control. qPCR was performed with a BioEasy SYBR-Green I Real Time PCR kit manual (Hangzhou Bioer Technology Co., Ltd., Hangzhou, China). The primers were as follows: RhoA, forward: 5′-CATCCCAGAAAAGTGGACTCCA-3′ and reverse 5′-CCT TGTGTGCTCATCATTCCG-3′, 103 bp; ROCK1, forward: 5′-GAATGACATGCAAGCGCAAT-3′ and reverse: 5′-GTC CAAAAGTTTTGCACGCA-3′, 113 bp; ROCK2, forward: 5′-GAAACAACTGGATGAAGCTAATGC-3′ and reverse: 5′-GTTTCAAGCAGGCAGTTTTTATCTT-3′, 150 bp; type-I procollagen, forward: 5′-TTCACCTACAGCACGCTTGT-3′, reverse: 5′-TTGGGATGGAGGGAGTTTAC-3′, 196 bp; type-III procollagen, forward: 5′-TTGAATATCAAACAC GCAAGGC-3′ and reverse: 5′-GGTCACTTTCACTGGTTG ACGA-3′, 201 bp; GAPDH, forward: 5′-GATGGGTGT GAACCACGAGAAA-3′ and reverse: 5′-ACGGATACATTG GGGGTAGGAA-3′, 330 bp. The RhoA, ROCK1, ROCK2, collagen type I and III amplification conditions were: Pre-denaturation at 95°C for 2 min, denaturation at 95°C for 20 sec, annealing at 58°C for 25 sec, and extension at 72°C for 30 sec, for a total of 45 cycles. The PCR products underwent electrophoresis and were scanned with a gel image analysis system [UV-2000; UNICO (Shanghai) Instruments Co., Ltd., Shanghai, China]. The intensity of RhoA, ROCK1, ROCK2, collagen type I and III was standardized to that of the GAPDH mRNA levels.

### Western blot analysis

Small sections of the atria of the rats were lysed. Protein was extracted and measured using a bicinchoninic acid (BCA) protein assay kit (Pierce Biotechnology, Inc., Rockford, IL, USA). Protein (~50 μg) was separated by 10% SDS-PAGE and transferred to polyvinylidene fluoride (PVDF) membranes. The membranes were blocked with 5% fat-free milk in Tris-buffered saline with Tween (TBST) buffer (20 mmol/l Tris-HCl, pH 7.5, 150 mmol/l NaCl and 0.05% Tween 20), and subsequently incubated with the following primary antibodies: polyclonal rabbit anti-rat RhoA (Santa Cruz Biotechnology, Inc., Santa Cruz, CA, USA), polyclonal goat anti-rat ROCK1 (Santa Cruz Biotechnology, Inc.), and polyclonal goat anti-rat ROCK2 (Santa Cruz Biotechnology, Inc.) at 4°C overnight. The mixture was washed and then incubated for 1 h with horseradish peroxidase (HRP)-conjugated secondary antibodies (Kirkegaard & Perry Laboratories, Inc., Gaithersburg, MA, USA). The membranes were developed using an enhanced chemiluminescence kit (Pierce Biotechnology, Inc.). Quantification of bands was performed by gel densitometry with a gel image analysis system (UVP, LLC, Upland, CA, USA). The phosphorylation level was normalized by total protein-band densitometry individually.

### Statistical analysis

The SPSS 17.0 statistical software package (SPSS, Chicago, IL, USA) was used for analysis. Data are presented as the mean ± standard deviation. All the groups were tested for normal distribution and equal variance. Differences among the three groups (at week 24) were assessed using one-way analysis of variance. P<0.05 was considered statistically significant.

## Results

At week 24, compared with the control rats, the levels of FBG, FINS, and HOMA-IR were significantly increased in untreated and fasudil-treated diabetic rats. No significant differences were identified in these indices between untreated and treated diabetic rats, suggesting that the 10 mg/kg/day fasudil administered had no effect on glucose metabolism and insulin resistance (Table I).

### Myocardial fibrosis

**Figure 1:**
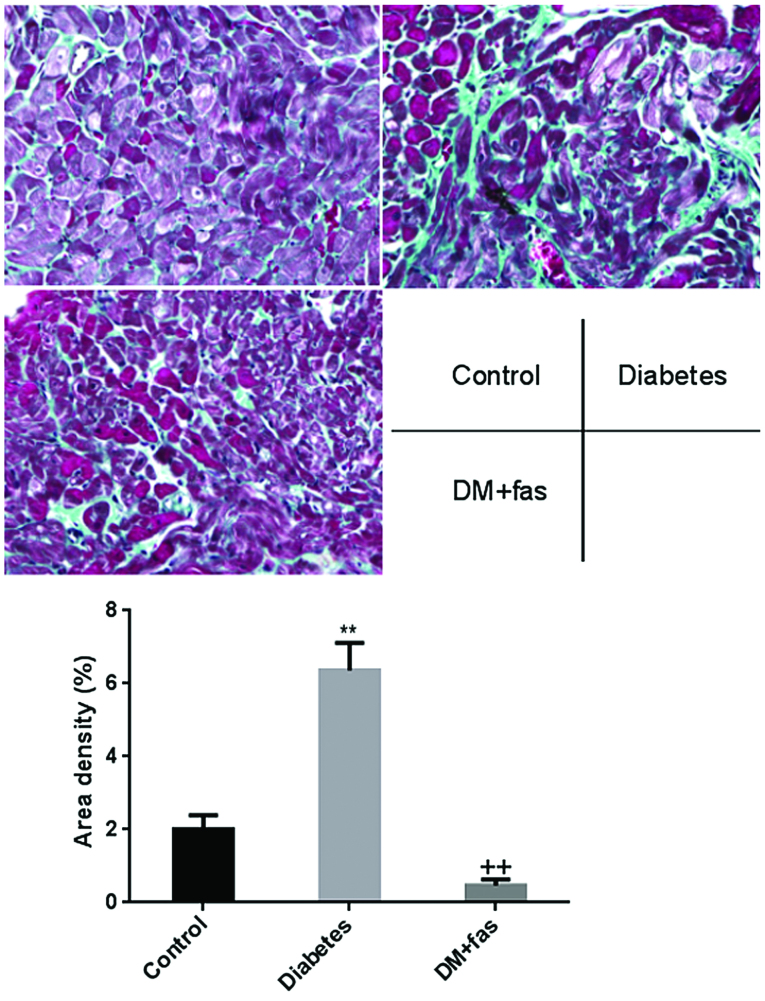
The deposition of collagen in the atrial interstitium in Masson-stained sections of the control, diabetic and diabetic rats treated with fasudil (DM+fas). Heart tissues from the diabetic group show focal regions of fibrosis (blue) in the interstitium (P<0.01 vs. the control and DM+fas groups). Magnification, ×400. The number of rats in each group was six.

Representative patterns of Masson staining demonstrating atrial fibrosis are shown in Fig. 1. The deposition of collagen in the atrial interstitium from untreated diabetic hearts was significantly increased compared with that observed in the control rats. However, treatment with fasudil significantly reduced the deposition of collagen.

### Gene expression

**Figure 2:**
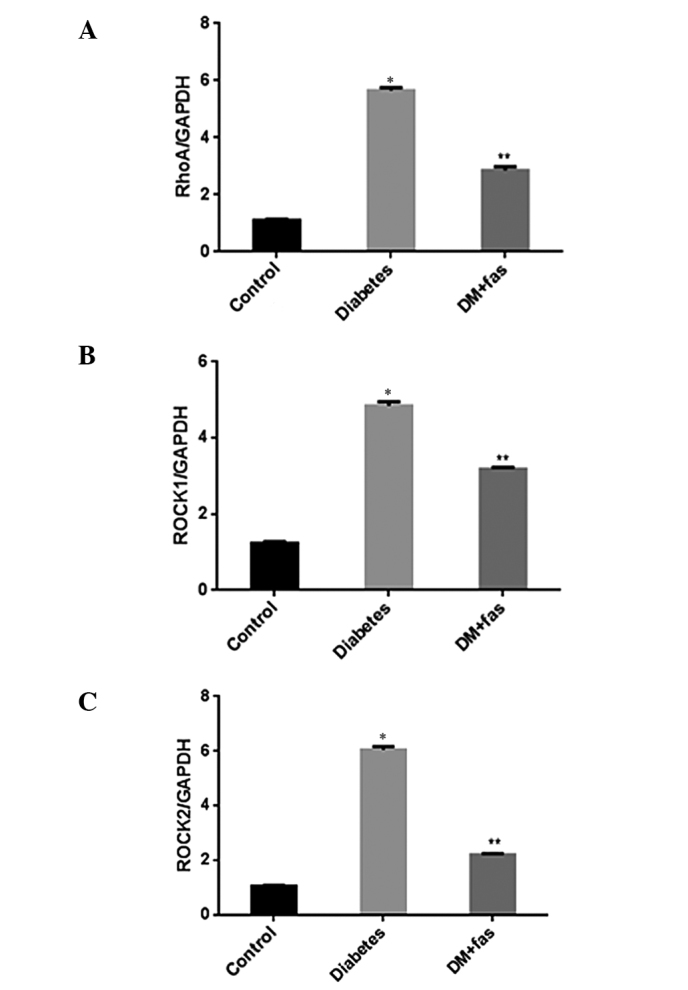
mRNA expression levels of (A) RhoA, (B) ROCK1 and (C) ROCK2, in untreated diabetic, control and DM+fas rats are evaluated by quantitative polymerase chain reaction. The intensities of RhoA, ROCK1 and ROCK2 were standardized to that of GAPDH. The number of rats in each group was six. The mRNA expression levels of RhoA, ROCK1 and ROCK2 were significantly increased in diabetic rats and decreased in DM+fas rats. ^*^P<0.01 vs. control rats,^**^P<0.01 vs. diabetic rats. DM+fas, diabetic treated with fasudil.

**Figure 3:**
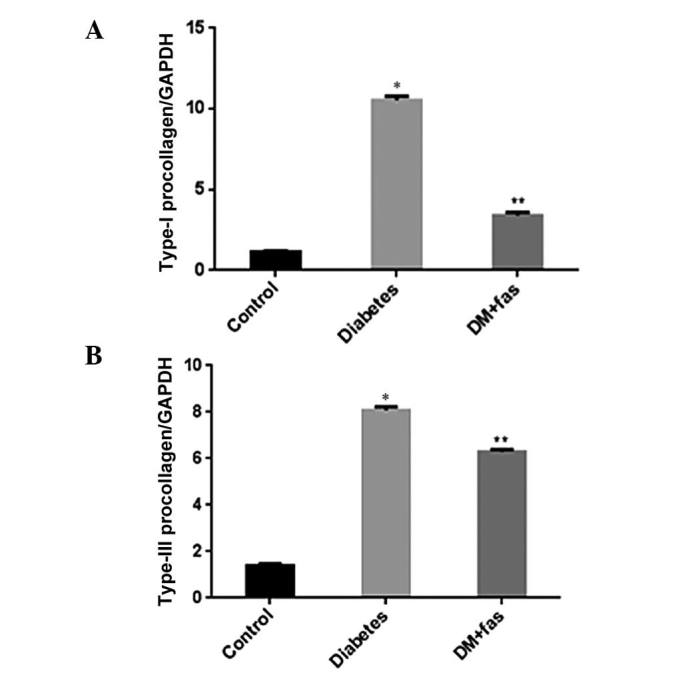
The mRNA expression levels of (A) type I and (B) type III procollagen in untreated diabetic, control and DM+fas rats was evaluated by quantitative polymerase chain reaction. The intensities of type I and III procollagen were standardized to that of GAPDH. The number of rats in each group was six. The mRNA expression levels of type I and III procollagen were significantly increased in diabetic rats and decreased in DM+fas rats. ^*^P<0.01 vs. control rats,^**^P<0.01 vs. diabetic rats. DM+fas, diabetic treated with fasudil.

Compared with the control rats, mRNA expression levels of RhoA, ROCK1 and ROCK2 (Fig. 2A–C) were significantly increased in the hearts of untreated diabetic rats. The upregulation of mRNA expression of RhoA, ROCK1 and ROCK2 was blocked by treatment with fasudil. mRNA expression of type I and III procollagen (Fig. 3A and B) was significantly upregulated in the hearts of the untreated diabetic rats, however, this expression was inhibited by treatment with fasudil. These results suggest that fasudil exhibited inhibitory effects on diabetes-induced gene upregulation of type I and III procollagen, RhoA, ROCK1 and ROCK2.

### Protein expression

**Figure 4:**
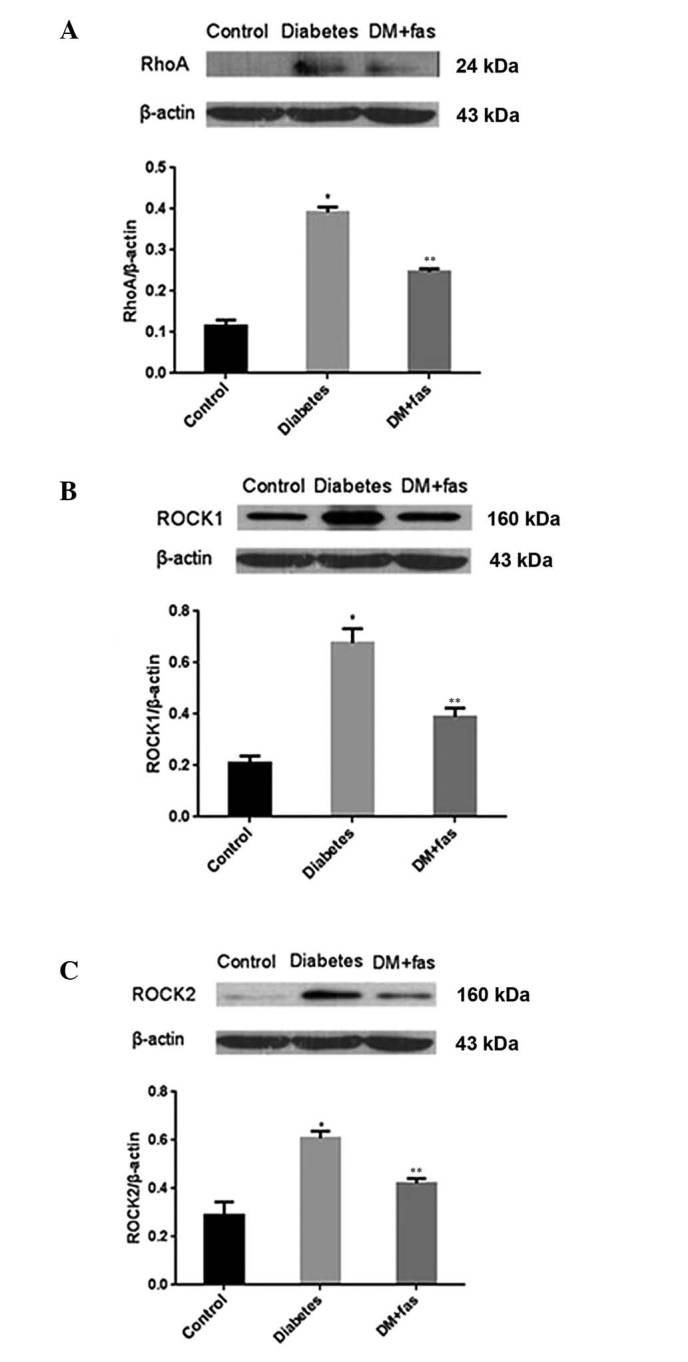
The protein levels of (A) RhoA, (B) ROCK1 and (C) ROCK2 in control, diabetic and DM+fas rats were evaluated by western blot analyses. The protein levels of RhoA, ROCK1 and ROCK2 were significantly increased in diabetic rats and decreased in DM+fas rats. ^*^P<0.05 vs. control rats, ^**^P<0.05 vs. diabetic rats. DM+fas, diabetic treated with fasudil.

The protein levels of RhoA (Fig. 4A), ROCK1 (Fig. 4B) and ROCK2 (Fig. 4C) were measured, and all three proteins were significantly enhanced in the hearts of diabetic rats compared with those of control rats. In diabetic rats, RhoA, ROCK1 and ROCK2 were attenuated by treatment with fasudil compared to the untreated diabetic rats. These results suggested that fasudil inhibited activation of the Rho/ROCK pathways.

## Discussion

Previous studies have shown that rats fed a HFD develop insulin resistance (14–15), and low-dose STZ is known to induce a mild impairment of insulin secretion (16). In the present study, the experimental rats fed HFD combined with low-dose STZ (30 mg/kg, once, i.p.) exhibited hyperglycemia and hyperinsulinemia. The results suggested insulin resistance in diabetic rats. Therefore, the present study successfully established a model of type 2 DM in the rat, and this model closely mimics the natural history and metabolic characteristics of type 2 DM in humans.

In the present study, the atria of untreated diabetic rats showed notable increases in RhoA/ROCK activity and marked atrial fibrosis as compared to that in the control rats. Treatment with fasudil hydrochloride hydrate significantly reduced RhoA/ROCK activity and atrial fibrosis. The inhibitory effect of fasudil on collagen fibers was regulated at least as far upstream as the transcriptional level, since treatment with fasudil suppressed the mRNA expression of type I and III procollagen. The findings are in agreement with the results of other previous studies (9–12,17).

The Rho family of proteins is a group of small guanosine triphosphate-binding proteins. ROCK is a downstream signaling molecule of RhoA that has been widely investigated. ROCK is activated by RhoA and phosphorylates the cytoplasmic MLC. Through this pathway, ROCK regulates several biological processes, including cell adhesion, chemotaxis and contraction. Inhibitors can inhibit ROCK activity through competitive binding to the adenosine triphosphate-binding site of the ROCK catalytic domain (18).

Fasudil and Y-27632 are non-isoform-selective ROCK inhibitors and equivalently inhibit ROCK1 and ROCK2. Furthermore, at higher concentrations, these ROCK inhibitors also inhibit other serine-threonine kinases, including protein kinase (PK) A and PKCU. Nevertheless, fasudil and its active metabolite, hydroxyfasudil, are more selective for ROCKs than other kinases, with hydroxyfasudil being slightly more selective than fasudil and Y-27632 (19). In the present study, the effect of restraining atrial fibrosis was associated with the RhoA/ROCK pathway.

The RhoA/ROCK pathway controls a wide variety of signal transduction pathways. ROCK1 has been shown to be a regulator of glucose homeostasis and insulin sensitivity *in vivo* (20). However, previous studies on the effects of ROCK inhibitors on glucose and lipid metabolism *in vivo* have yielded conflicting results. The different conclusions obtained among these studies may be due to the use of different inhibitors, doses, treatment times and animal models. Kikuchi *et al* (9) found that a low-dose of fasudil (30 mg/kg/day) did not affect glucose and lipid metabolism. However, high-dose fasudil (100 mg/kg/day) ameliorated the metabolic disorder. Chronic treatment of obese db/db mice with fasudil (10 mg/kg/day) has no effect on BG levels and blood pressure (10). Komers *et al* (21) found that the fibrosis inhibitory effect of fasudil was without reduction of blood pressure. In the present study, a low-dose of fasudil (10 mg/kg/day) exhibited no effects on BG, insulin resistance nor blood pressure in diabetic rats. Thus it appears that the effect of fasudil was independent of blood pressure and glycemic control.

The present study showed that RhoA/ROCK was involved in atrial fibrosis, and fasudil hydrochloride hydrate ameliorated atrial fibrosis through the RhoA/ROCK pathway in rats with type 2 diabetes. Inhibition of the RhoA/ROCK pathway may therefore be a novel therapeutic target for the prevention of atrial fibrosis. Additionally, the RhoA/ROCK pathway is involved in atrial fibrosis in rats with type 2 diabetes. However, there were certain experimental limitations in this study. First, to improve understanding of the mechanism of action involved, cell culture studies are required to verify the cause and effect associations with regards to the role of these signaling molecules in diabetic cardiac fibrosis. Second, it is known that atrial fibrosis is associated with significantly increased oxidative stress and inflammation. Therefore, the characteristics of oxidative stress and inflammation in the heart should be described. Third, the small number of rats in the present study, may limit the statistical power and results. Fourth, as the conclusion of fasudil exhibiting no effect on blood pressure was obtained from the preceding studies, measures should be taken to further verify the result. The treatments used to prevent the development and progression of atrial fibrosis are not fully confirmed, and further clinical trials are required to corroborate the conclusions of the present study.

## Figures and Tables

**Table I:** The effects of fasudil on glucose metabolism parameters in rats at week 24.

Group	Control	Diabetic	DM+fas
FBG (mmol/l)	5.65±0.23	19.57±0.71^a^	20.10±0.61^a^,^b^
FINS (mU/l)	33.95±2.51	52.53±5.47^a^	47.08±2.90^a^,^b^
HOMA-IR	8.50±0.64	44.13±3.25^a^	41.93±2.51^a^,^b^

Differences among the three groups were assessed using one-way analysis of variance. The levels of FBG, FINS and HOMA-IR in the diabetic and the DM+fas groups were significantly increased compared to those in the control group, while no difference was identified between the diabetic group and the DM+fas group.

a P<0.05 compared with the control group,

b P>0.05 compared with the diabetic group. The number of rats in each group was six. Data are presented as the mean ± standard deviation.

DM+fas: diabetic rats treated with fasudil; FBG, fasting blood glucose; FINS, fasting insulin; HOMA-IR, homeostasis model assessment for insulin resistance, calculated as FBG (mmol/l) x FINS (mU/l)/22.5.
